# How to conduct an individual participant data meta-analysis in response to an emerging pathogen: Lessons learned from Zika and COVID-19

**DOI:** 10.1017/rsm.2025.10029

**Published:** 2025-11-03

**Authors:** Lauren Maxwell, Priya Shreedhar, Laura Merson, Brooke Levis, Thomas P. A. Debray, Valentijn Marnix Theodoor de Jong, Ricardo Arraes de Alencar Ximenes, Thomas Jaenisch, Paul Gustafson, Mabel Carabali

**Affiliations:** 1 Heidelberger Institut für Global Health, Universitätsklinikum Heidelberg, Heidelberg, Germany; 2 Ecraid Foundation, Provinciehuis, Utrecht, the Netherlands; 3 ISARIC, Pandemic Sciences Institute, University of Oxford, Oxford, UK; 4 Public Health Department, Institut Pasteur de Dakar, Dakar, Senegal; 5 Centre for Clinical Epidemiology, Lady Davis Institute for Medical Research, Jewish General Hospital, Montreal, QC, Canada; 6 Julius Center for Health Sciences and Primary Care, UMC Utrecht, Utrecht University, Utrecht, the Netherlands; 7 Data Analytics and Methods Task Force, European Medicines Agency, Amsterdam, the Netherlands; 8 Avenida Moraes Rego, Cidade Universitária, Recife, Brazil; 9 Department of Epidemiology, Center for Global Health, Colorado School of Public Health, Aurora, CO, USA; 10 Heidelberger Institut für Global Health, Universitätsklinikum Heidelberg, Heidelberg, Germany; 11 Department of Statistics, The University of British Columbia, Vancouver, BC, Canada; 12 Department of Epidemiology, Biostatistics and Occupational Health, School of Population and Global Health, Faculty of Medicine and Health Sciences, McGill University, Montreal, QC, Canada

**Keywords:** ELSI barriers, emerging pathogens, FAIR principles, individual participant data meta-analysis, infectious diseases, measurement error

## Abstract

Sharing, harmonizing, and analyzing participant-level data is of central importance in the rapid research response to emerging pathogens. Individual participant data meta-analyses (IPD-MAs), which synthesize participant-level data from related primary studies, have several advantages over pooling study-level effect estimates in a traditional meta-analysis. IPD-MAs enable researchers to more effectively separate spurious heterogeneity related to differences in measurement from clinically relevant heterogeneity from differences in underlying risk or distribution of factors that modify disease progression. This tutorial describes the steps needed to conduct an IPD-MA of an emerging pathogen and how IPD-MAs of emerging pathogens differ from those of well-studied exposures and outcomes. We discuss key statistical issues, including participant- and study-level missingness and complex measurement error, and present recommendations. We review how IPD-MAs conducted during the COVID-19 response addressed these statistical challenges when harmonizing and analyzing participant-level data related to an emerging pathogen. The guidance presented here is based on lessons learned in our conduct of IPD-MAs in the research response to emerging pathogens, including Zika virus and COVID-19.

## Highlights

### What is already known?

- Individual participant data meta-analyses (IPD-MAs) that harmonize and analyze participant-level data from primary studies are central to the rapid research response to epidemics of emerging pathogens.
- IPD-MAs have several advantages over aggregate data meta-analysis, such as the inclusion of additional participants and longer follow-up time; evaluation of confounding, interaction effects, and nonlinear effects; minimization of publication and reporting bias; and the identification of groups and/or individuals at the highest risk of severe outcomes.
- IPD-MAs are also resource intensive and may require large, international collaborations with resources to support the required project management and harmonization inputs.

### What is new?

- A key learning from our experience setting up and implementing Zika and COVID-19-related IPD-MAs is that the participant-level data collected from observational studies launched in response to an emerging pathogen can differ importantly in terms of quality and usability compared to data from a randomized controlled trial (RCT) or data related to well-described infectious or chronic diseases.
- In EID response, where evidence is scarce and concurrent data collection efforts proliferate rapidly, the alignment and harmonization of inclusion criteria, outcomes, and protocols facilitated by prospective IPD-Mas (PMAs) can enhance both the speed and relevance of IPD-MAs.
- Researchers undertaking an IPD-MA in the context of an emerging pathogen face additional barriers to data collection or face sharing, harmonization- and analysis-related challenges, due to the evolving knowledge of the emergent pathogen and resources allocated for it.
- To help address these challenges, in this review, we present details of the different steps required to develop and implement an IPD-MA that consists of data from longitudinal observational studies and surveillance data collected as part of the research and public health response to an emerging pathogen.

### Potential impact for RSM readers

- As IPD-MAs are resource intensive and often are large collaborations, the best practices in relation to IPD-MA project management, data cleaning, and data harmonization that we provide in this review are likely to be able to help aid the more efficient conduct of an IPD-MA in any discipline.
- We also address ethical and data-sharing concerns in relation to the collection and harmonization of participant- and study-level data in the research response to an emerging pathogen which are not often talked about and are useful to understand for those looking to conduct an IPD-MA of an emerging pathogen.

## 1 Introduction

Participant-level data is an essential resource for informing the public health and research response to an emerging pathogen.^1^ ^,^ ^2^ Individual participant data meta-analyses (IPD-MAs), which harmonize and analyze participant-level data from related studies, have several advantages over aggregate data meta-analyses (AD-MAs), which combine effect estimates and their uncertainty.^3^ These include the ability to (1) consider both study- and participant-level characteristics simultaneously, which facilitates evaluation of confounding, interaction effects, and nonlinear effects; (2) identify those who may benefit the most from treatment; (3) minimize publication and reporting bias by including both published and unpublished studies; and (4) include, for IPD-MAs of longitudinal data, additional participants and follow-up time beyond those included in related publications.^3^ ^–^ ^6^ Despite these advantages, IPD-MAs are resource intensive and generally require large, international collaborations with resources to support the required project management and harmonization.^6^ ^,^ ^7^

### 1.1 The need for rapid, coordinated evidence generation in EID response

Inference during an EID must balance speed, research and data integrity, and equity.^8^ In the research response to an emerging infectious disease (EID), AD-MAs can generate evidence for policy and practice recommendations early in the pandemic. In the COVID-19 pandemic, some early meta-analyses led to important changes in clinical practice.^9^ That said, other AD-MAs were skewed by inaccurately or inadequately reported or biased trials.^10^ While both IPD- and AD-MAs are affected by the quality of included studies, IPD-MAs have a number of advantages over AD-MAs for investigating research and data integrity issues,^11^ which are more likely to affect studies that respond to EIDs than studies conducted outside of EIDs.^12^ IPD-MAs include a data cleaning step where outliers will be interrogated and research integrity issues, like those that affected ivermectin-treatment-related retractions during COVID-19 response,^13^ may be identified. IPD-MAs can be used to address risk of bias (RoB)concerns in primary studies, assuming such changes are possible within the original dataset.^14^ For example, in contrast to an AD-MA, infection or outcome definitions that may have evolved during the epidemic can be aligned in the IPD-MA to address selection misclassification biases.

### 1.2 Role of IPD-MAs in public health emergencies

While AD-MAs will be deployed more rapidly, IPD-MAs are needed to understand subgroup and interaction effects and evaluate clinical risk prediction models,^15^ which facilitate more targeted approaches to diagnosis, prevention, and treatment. Subgroup analyses are difficult in AD-MAs, which may not provide sufficient data to consistently apply subgroup criteria to assess variability in treatment effectiveness or vaccine efficacy across patient demographics or disease severities. IPD allow for the standardization of exposure, confounder, and outcome measures across studies, addressing spurious sources of heterogeneity that cannot be addressed in an AD-MA other than through study inclusion criteria or sensitivity analyses. IPD-MA can be updated with new data, and diagnostic and confounder criteria can be adjusted as researchers gain familiarity with the EID, supporting timely, data-driven public health decision-making. IPD facilitates more effective handling of missing data than an AD-MA by enabling multiple imputation of study- and participant-level data which can reduce bias, increase sample size, and increase the reliability of the findings.^16^ ^,^ ^17^ Perhaps most importantly, IPD enables the direct assessment of interactions between treatments and participant characteristics and the consistent consideration of nonlinearities in the relation between key covariates, which can help explain differences in infection, vaccine efficacy, or disease. IPD-MA allow for one-stage and two-stage analyses. One stage-analysis uses a more exact statistical likelihood, which benefits inference when included studies have few participants or few participants experience the outcome of interest,^18^ which may be the case for an EID, especially early in the outbreak.

### 1.3 Operational challenges and considerations

Participant-level data collected from observational studies launched in response to an epidemic of an emerging pathogen differ importantly from data collected for a randomized controlled trial (RCT) or data related to well-described infectious or chronic diseases (e.g., HIV, cardiovascular disease).^19^ The guidance included in this tutorial stems from our groups’ leadership of several major IPD-MA initiatives during Zika virus (ZIKV) and COVID-19 response. These include the Zika Brazilian Cohorts’ Consortium IPD-MA of Brazilian cohorts of women and children^20^; the World Health Organization (WHO)-moderated ZIKV Individual Participant Data (IPD) Consortium, a global initiative that brought together all ZIKV-related cohorts of pregnant women and their infants and children^21^; the COVID-19-mortality-related IPD-MA of data from clinical prediction models from the CAPACITY-COVID Consortium,^22^ and the International Severe Acute Respiratory and Emerging Infection Consortium (ISARIC) effort to facilitate the prospective harmonization of COVID-19-related IPD.^23^

Researchers undertaking an IPD-MA in the context of an emerging pathogen face additional barriers to data collection (e.g., due to changes in diagnosis and reporting), sharing, harmonization and analysis, including evolving knowledge of the emergent pathogen and disease manifestations^19^; the premium placed on data generated during epidemic response, which disincentivizes sharing^24^; and the gap between funding and cases, which can result in studies missing the epidemic.^25^ The heterogeneity of diagnostic tools and algorithms and the lack of a gold standard are major challenges in the research response to emerging pathogens, as demonstrated by the variable accuracy of diagnostics deployed in ZIKV^26^ and COVID-19^27^ response.

To facilitate the trust needed for data sharing and ensure that data are interpreted correctly, the groups that collected the data should be involved in every step of the IPD-MA process, from development to dissemination.^28^ This requires funding to be made available to the contributing groups, for example, to maintain an operational team to make necessary changes to the datasets to harmonize the variables. Harmonization should be paired with applying the findable, accessible, interoperable, and reusable (FAIR) principles for data stewardship^29^ and address ethical and benefit-sharing concerns.^8^ In addition, the IPD-MA should consider incorporating perspectives from the studies’ source communities to ensure that the investment in the IPD-MA and related results leads to meaningful advances in clinical practice for the data-contributing communities.^30^

### 1.4 Scope and purpose of this tutorial

In this tutorial, we present the steps required to develop and implement an IPD-MA that consists of data from longitudinal observational studies and surveillance data collected as part of the research and public health response to an emerging pathogen. We also discuss key barriers and paths forward when conducting an IPD-MA of an emerging pathogen so that the results can provide timely, actionable information for clinical or public health practice. Additionally, we review approaches to harmonization, statistical issues, and political, ethical, administrative, regulatory, and legal (PEARL) barriers to sharing data^31^ for an IPD-MA of an EID to facilitate future IPD-MAs.

## 2 Undertaking an IPD-MA in the context of an EID

**Figure 1:**
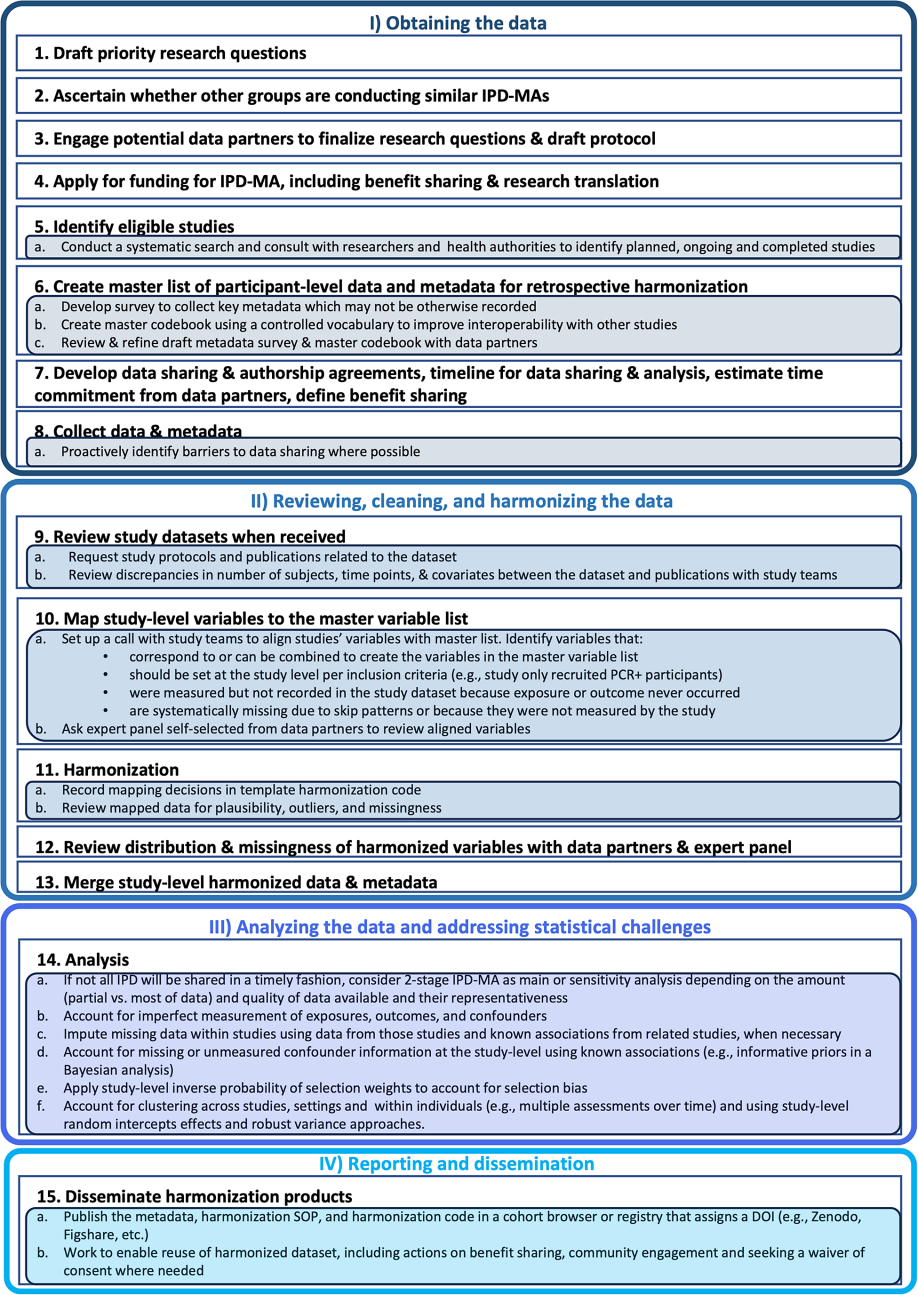
Design and implementation of IPD-MAs of an emerging pathogen, from the research question to the creation of the analytic dataset and results dissemination, adapted from Maelstrom Research Group.^32^

Figure 1 provides an overview of the IPD-MA process from the identification of research questions through dissemination. To facilitate data reuse and risk communication in the presence of uncertainty, IPD-MAs of data collected in the research response to an EID should additionally include (1) application of the FAIR principles to participant-level data and study-level metadata to facilitate reuse of the data beyond the initial IPD-MA; (2) ascertaining community preferences for data sharing and risk communication in the presence of uncertainty; and (3) taking steps needed to enable the reuse of the harmonized datasets for public-health-relevant purposes other than the objectives of the primary research studies (per the laws and guidelines at the investigators’ institutions), including community engagement and applying for waivers of consent where broad consent for future use was not obtained. Harmonization standard operating procedures (SOPs) should be based on best practice, well documented, and consistently applied to streamline work and ensure consistency over time.^32^ The inclusion of OMICs or high-dimensional imaging data warrants the consideration of additional harmonization, statistical, and ethical concerns beyond those described here.

### 2.1 Ascertain whether other groups are conducting similar IPD-MAs

EID outbreaks’ urgency and global scale lead to heightened interest from multiple research groups, increasing the likelihood of concurrent IPD-MAs. Given the limited availability of high-quality participant-level data early in an EID response, coordinated efforts across related IPD-MAs are especially critical to avoid duplication, maximize the value of scarce data, and produce timely, policy-relevant evidence. Identifying and describing concurrent, related IPD-MAs is a much-needed first step before launching another IPD-MA in EID response. We conducted a rapid systematic review to identify ongoing or completed COVID-19-related IPD-MAs. We found 31 IPD-MAs, many of which had overlapping study designs, populations, exposures, and outcomes of interest, representing a missed opportunity for collaboration.^33^ IPD-MA protocols are hosted in different databases, including International Prospective Register of Systematic Reviews (PROSPERO), Open Science Framework (OSF), preprint servers, or publication databases, including Web of Science or Ovid (Medline), which complicates their identification. Search for related protocols and post your own IPD-MA protocol as widely, with as much detail, and as soon as possible, to conserve limited resources by encouraging cross-initiative collaborations.

### 2.2 Convene researchers to foster collaboration

While most IPD-MAs are based on existing data and can be considered retrospective studies, planning an IPD-MA ahead of primary data collection, including defining the research objectives, protocol, and case report form (CRF), facilitates later harmonization efforts.^34^ Following learnings from the ISARIC’s rapid development of a FAIR-by-design electronic case report form (eCRF) in the COVID-19 response, a team of researchers has developed BRIDGE (Bioresearch Integrated Data Tool Generator), a software approach to supporting the cooperative development of FAIR-by-design eCRFs in the research response to emerging pathogens.^35^ ^,^ ^36^ By employing an open, collaborative process, the BRIDGE approach to eCRF development leverages global expertise in confronting the EID while providing an open-source platform for data capture that can be modified to suit the needs of local researchers.

If no other IPD-MA efforts are identified, snowball sampling can be used to bring together researchers working in the field to share research protocols and CRFs to facilitate later harmonization efforts. Prospective IPD-MAs (PMAs), where inclusion criteria, protocols, eCRFs, and analysis plans are defined in advance,^37^ facilitate harmonization of participant-level data during epidemic response. PMAs ensure incoming study data are aligned for synthesis, facilitating harmonized IPD collection, which is especially useful when outcomes are rare or inconsistently reported.^37^ Given the urgency and uncertainty surrounding an EID, the chances of multiple, overlapping studies addressing the same research questions are higher than in more stable research contexts. Thus, the PMA approach not only reduces duplication and research waste but also maximizes the utility of scarce and emerging data streams through coordinated prospective planning.

Still, the heterogeneity of diagnostic algorithms, protocols, and definitions of studies that are not engaged in a prospective IPD-MA can also be helpful in the research response to emerging pathogens. For example, in the ZIKV response, initial cohort studies were limited to symptomatic pregnant women, but later studies found that only around 30% of cases were symptomatic.^38^ ^,^ ^39^ Similarly, in ZIKV, very few studies assessed infant and child outcomes in children without human-observable signs of congenital ZIKV syndrome at birth.^40^ ^,^ ^41^ As the children from ZIKV-affected pregnancies developed, researchers realized that depending on human observable outcomes was insufficient, and specific outcomes that manifested when children began language development may have only been observable by magnetic resonance imaging (MRI) at birth.^41^ In both these examples, the scientific community benefited from the heterogeneity of protocols, some of which included asymptomatic pregnant women or followed children who were exposed to ZIKV in utero but where there was no evidence of congenital Zika syndrome (CZS) at birth. Despite prospective coordination of efforts, in the research response to an EID, IPD-MAs will include both retrospective and prospective components as groups react differently to emerging information about the pathogen. The IPD-MA budget, process, and statistical plan must reflect this complexity.^19^

### 2.3 Develop the IPD-MA research protocol, data management, and statistical analysis plans

As with any IPD-MA, the protocol for identifying studies and creating the analysis dataset during the research response to an EID should be developed in coordination with data contributing groups and published according to the Preferred Reporting Items for Systematic review and Meta-Analysis Protocols (PRISMA-P) guidelines.^42^ Similarly, the statistical analysis plan (SAP) should be developed with data contributing groups and published in an established registry (e.g., Open Science Foundation [OSF], Zenodo) before beginning analysis. In the research response to an emerging pathogen, new information may lead to significant departures from the original SAP, which should be disclosed in related publications. Most IPD-MAs are limited to data from randomized clinical trials of well-known exposures. In the research response to an emerging pathogen, study quality may decrease as rapidity takes precedence over scientific rigor. The SAP needs to include a comprehensive approach to addressing the complicated forms of measurement error that are likely to affect the IPD-MA.

### 2.4 Identify and recruit eligible studies

When a new pathogen emerges, existing cohorts and trials are repurposed to study the risk and effects of infection.^43^ ^,^ ^44^ Identifying these cohorts and trials and organizing a joint research response is a crucial challenge. Cohorts that publish research protocols and initial analyses in a peer-reviewed journal can be identified through a (rapid) literature review or by consulting existing reviews; however, this process is slow and cannot identify smaller cohorts. Trials can be identified through structured searches of clinical trial registries.^45^ Additional cohorts and trials may be identified through reviewing preprint servers (e.g., medRxiv), consultation with researchers who are leading related cohorts or with regional and international funding and implementing organizations (e.g., WHO and regional offices, Ministries of Health), and disseminating the call for studies to participate in the IPD-MA through social media and academic networks. While PMAs are especially important in EID response, where observational and intervention studies emerge rapidly in response to the outbreak, the need to repurpose existing cohorts or surveillance studies, which are often the first to detect emergent cases,^46^ poses challenges since such studies lack prospective coordination and use variable protocols and data collection schema.

### 2.5 Develop a budget and secure funding

IPD-MAs are resource-intensive, large, multinational, and multidisciplinary collaborations that require a well-organized and stable team.^6^ ^,^ ^7^ In both routine IPD-MAs and those conducted in EID response, researchers approach funders after defining the core objectives of the IPD-MA and securing the participation of at least several study investigators. In addition to groups that fund IPD-MAs conducted outside of EID response, IPD-MAs of emerging pathogens may also be funded by consortia focused on data sharing in epidemic response (e.g., the Global Research Collaboration for Infectious Disease Preparedness [GLOPID-R]).

Project management, collection of relevant metadata, and harmonization of participant-level data are the most resource-intensive components of IPD-MAs and necessitate a larger proportion of the budget for an IPD-MA of an emerging pathogen where diagnostics and laboratory protocols are evolving, outcome and exposure definitions are under debate, individual study protocols and metadata change as the understanding of the pathogen evolves, and community-developed standards for participant-level data (e.g., Clinical Data Interchange Standards Consortium [CDISC]-Clinical Data Acquisition Standards Harmonization [CDASH])^47^ may not yet exist. This evolution has a two-fold impact on the budget. First, for the original study, primary studies on EIDs must adapt to changing information on the pathogen, which requires additional follow-up time, new ascertainment strategies, a shift in study sites, or the inclusion of additional covariates beyond what was initially envisaged, leading to underfunded primary data collection, which then results in delayed sharing or reduced recruitment or follow-up time, affecting the richness of data shared. Second, funding-related delays in data sharing or quality issues may derail established timelines for the IPD-MA harmonization, cleaning, and analysis work. Reusing datasets generally means additional work for the research group that has generated the data in the first place, and funding their time should be prioritized where possible. We developed a budget template to facilitate the estimation of time and funding from the core management team, subject matter experts, and primary research study teams.^48^ ^,^ ^49^ Ensuring the EID IPD-MA management and harmonization team has alternative projects can help ensure continuity when faced with delays as primary studies balance competing demands for their time and expertise.

### 2.6 Engage study investigators

Partnership with the groups that conducted included studies is a central requirement for any IPD-MA, but is especially important in IPD-MAs for EIDs, where rapid, meaningful engagement facilitates the close interaction needed to clean and interpret the data. The primary study teams have the necessary expertise on the emerging pathogen or closely related pathogens. They can speak honestly about modeling the heterogeneity in background risk and measurement, central to informing imputation and outcome models. In the early stages of understanding an EID, the nuances of local or regional case definitions and changing enrollment procedures, diagnostic practices, and treatment protocols may not be fully documented and can only be clarified through active investigator engagement.^19^ As highlighted during the planning for a coordinated Oropuche research response, meaningful engagement of investigators from affected regions and institutions is critical not only for accurate data interpretation but also for building trust in IPD-MA results and resulting interventions.^50^

Investigator engagement can be facilitated through an initial email with a frequently asked questions (FAQ) document, followed by a call to help build the personal relationships needed to support active investigator engagement in the IPD-MA. We have developed template emails, a sample FAQ document, and investigator contact SOPs to facilitate this process as part of an IPD-MA Toolkit.^48^ ^,^ ^49^ Participation in the IPD-MA represents a significant investment of investigator time to collect descriptive metadata, finalize data cleaning efforts, and support the harmonization and analysis efforts.

Expert working groups led by investigators from participating studies can guide the selection of participant-level variables for harmonization, required metadata, and a preferred standard for participant-level clinical and epidemiological data (e.g., CDASH, Systemized Nomenclature of Medicine—Clinical Terms [SNOMED-CT], the Observational Medical Outcomes Partnership [OMOP] common data model [CDM])^47^ ^,^ ^51^ ^,^ ^52^ or metadata (e.g., schema.org, Data Documentation Initiative [DDI] codebook.org).

As in the Zika Brazilian Cohorts Consortium (ZBC)- and ZIKV IPD Consortia, a continuous interaction between the statistical analysis group, primary study teams, and expert working groups is critical for accurately interpreting data and defining future analyses.^20^ ^,^ ^53^ Expert working groups are vital for IPD-MA conducted in the context of EIDs where exposure and outcome definitions may not yet be established, as was the case for CZS or long COVID. The IPD-MA should minimize the burden on investigators’ time, fund the participation of primary studies in the IPD-MA where possible, and create opportunities for forms of benefit sharing beyond authorship, including capacity building and longer-term mentorship opportunities.

### 2.7 Formalize collaboration and set expectations

Studies or surveillance systems that contribute data to the IPD-MA are generally asked to sign a letter of intent or another type of participation agreement and may want to codify authorship or intellectual property agreements. The participating institutions can define the terms of collaboration and may not require legally binding contracts. The IPD-MA Toolkit we developed contains a template non-legally binding letter of agreement.^48^ ^,^ ^49^ Requiring institutional rather than investigator-level sign-off led to important delays in the ZIKV IPD Consortium work. While some institutions may require institutional sign-off, following institution-specific guidelines rather than requiring all data partners to obtain institutional signatures would facilitate the earlier engagement of studies where the investigator’s signature is sufficient.

In the context of EIDs, clearly defining authorship criteria and agreeing on publication timelines early in the collaborative process becomes even more important.^50^ Data from EID response efforts are more likely to be relevant for high-impact publications and may be perceived by contributing investigators as especially valuable for academic promotion, funding, and recognition. This can heighten data-sharing-related concerns unless transparent expectations and fair benefit-sharing mechanisms are established from the outset.^54^ Explicit authorship policies and open communication regarding the publication process can help address these concerns and strengthen trust among collaborators. They may be especially important when data sharing is between researchers in high-income countries, organizing the IPD-MA, and researchers based in LMIC who have collected the data, and where ID outbreaks are more prevalent.^8^ ^,^ ^55^

### 2.8 Address ethics concerns

IPD-MAs themselves may generally qualify for a waiver of ethics review beyond the study’s initial ethics approval if the analysis is limited to reusing de-identified data from studies to fulfil the same objectives for which participants provided their initial consent. IPD-MAs may limit inclusion to research studies that have obtained participant-informed consent and can provide documentation of ethics committee review. A waiver of consent for the reuse of data or an exemption from ethical review, depending on laws and norms around data reuse, can be obtained to support the inclusion of surveillance, clinical care, population registry, or other public health data in an IPD-MA where participant consent for research was not obtained. While ERCs generally adopt expedited review procedures for EID-related research during an outbreak or epidemic,^56^ ^,^ ^57^ applying for a waiver of consent will likely prolong the IPD-MA timeline.

### 2.9 Share participant-level data

There are myriad barriers to sharing data, including study investigators’ need to first publish relevant study-specific results, legal barriers, and underfunded work to curate data and collect metadata.^31^ ^,^ ^58^ ^,^ ^59^ Some studies, particularly in the early stages of an outbreak, are self-funded using institutional resources, repurposed project funds, or investigators’ personal funds, which enables rapid study initiation but poses challenges for sustaining the work and for investing in additional unfunded data and metadata curation activities. Benefit-sharing, e.g., including data contributors as authors on resulting publications and through financial support for study staff time and capacity building, may help facilitate data sharing. Following best practice by closely involving data contributors in all phases of the IPD-MA, from funding to publication, including in IPD-MA design, funding applications, the development of timelines, and the harmonization and analysis processes, may also engender investigator support for the IPD-MA.

Neither harmonization nor analysis can be conducted without data. As such, the IPD-MA coordination team should encourage investigators to share de-identified data early to enable the harmonization process to begin while investigators continue to work on their main papers, with the understanding that the publication of those primary papers will take precedence over the IPD-MA itself. Ask investigators when they can share participant-level data, whether they prefer harmonization to be done in house or by the coordination team, and what data will be shared if they do not intend to share the entire de-identified dataset. Trust, open communication, and written, enforceable agreements can support sharing data for harmonization with the understanding that an individual study’s data will not be used for the IPD-MA if they disagree with the timeline. In an IPD-MA conducted in response to an EID, data will become available at different times, and the IPD-MA should conduct harmonization as data become available rather than waiting for all data to ensure progress toward a cleaned, harmonized dataset.

Waiting to share data until after study teams have published all their key papers can mean that harmonization, which can take several years, does not begin until after the epidemic. Timely sharing of participant-level data and the extraction of the metadata needed to reuse the data are central challenges for IPD-MAs in the research response to an EID. In contrast to well-described IDs and chronic diseases, sharing data generated in the research response to an emerging pathogen may be perceived as a greater cost to investigators who may execute studies without dedicated funding and generate important benefits, including high-impact publications and other forms of professional recognition through those data. In addition to ensuring that benefit sharing is meaningfully addressed in the context of the IPD-MA, funders should require rapid data sharing in all relevant funding calls, including clear language on timelines, expectations for interim and final data release, and reference to trusted repositories and the FAIR and collective benefit, authority to control, responsibility, and ethics (CARE) principles.^59^ They should also ensure researchers have appropriately budgeted for and otherwise fund the infrastructure and staffing required to curate, de-identify, and prepare data for reuse as part of the initial study award, and provide supplemental support for generating FAIR-by-design, harmonized study data and metadata during public health emergencies.^59^ Funders can further accelerate timely sharing by supporting machine-actionable data management plans, harmonization tooling, and coordinated legal frameworks, including preapproved templates for data use and transfer agreements.^59^

To ensure that the cross-study dataset can be delivered as soon as possible during the research response to an emerging pathogen, the IPD-MA team may want to work with investigators to establish a time after which the IPD-MA will not accept further data or otherwise determine how to proceed when some, but not all expected data are received. For the sake of trust and transparency, expectations for how data sharing relates to authorship are best determined through consensus at the start of the consortium. When consensus is impossible, an online survey may help establish the Consortium’s preferences for whether and how sharing data links to benefit sharing, including authorship. The IPD-MA toolkit we developed contains an example of a survey that was implemented to determine authorship preferences of the ZIKV IPD-MA Consortium.^48^ ^,^ ^49^ The information generated by IPD-MAs of an emerging pathogen can be critical for defining health policies. Therefore, the earlier this information is shared with health authorities, the greater the potential benefit to the population.^20^

Defining what the barriers are to data reuse is central to addressing those barriers. If the barriers are related to legal, ethical, or institutional concerns related to data transfer rather than to benefit sharing concerns, investigators can work toward the adoption of approaches to remote harmonization, like those developed by the Maelstrom group,^32^ and conversion to CDMs like Observational Medical Outcomes Partnership (OMOP)^52^ to enable federated reuse of participant data without moving or otherwise directly accessing the data.

### 2.10 Consider a two-stage IPD-MA when data sharing is not possible

When data sharing timelines are not aligned with harmonization and analysis goals, the IPD-MA team can consider a two-stage IPD-MA.^60^ The primary study investigators can then perform standardized analyses using template code provided by the IPD-MA team to generate relevant effect measures and their uncertainty (e.g., log hazard ratio and standard error). Estimates are then combined with the corresponding estimates from other studies in the second stage. This way, studies that cannot share IPD within the agreed-upon timeline due to logistical, legal, or ethical challenges can still participate in the IPD-MA. Federated data analysis, a privacy-by-design approach to participant-level data reuse where participant-level data are reused without being directly accessed, is generally not recommended given the high level of heterogeneity expected in IPD-MAs of EIDs, unless measurement error is explicitly addressed through penalization of sources of site-level heterogeneity.

### 2.11 Transfer data

Investigators are asked to share de-identified data, where direct identifiers, variables that uniquely identify a participant without being combined with other variables, and indirect identifiers, variables that can uniquely identify an individual when used in combination with other variables^61^ and that are not needed for analysis, are removed before the secure transfer of the data to the harmonization team. Special care is needed to ensure the de-identification of longitudinal data where the date variables are “connected.” For example, the date when a test sample from a severely ill patient was identified as positive and the date of hospitalization for the patient are connected. To minimize the potential for re-identification, researchers can present connected dates as the relative number of days from the reference date (e.g., date of admission or date of case identification).

Variables that can be important to the analysis, e.g., birth date to estimate effects of seasonal environmental exposures, may be coarsened (e.g., reduced to the month of birth) to remove the potential for using that variable in combination with other variables to identify a participant. Alternatively, the team can add a plausible, randomly generated number of days to the date of birth to protect participant privacy while retaining the variable’s original format. Data should only be shared between studies and the IPD-MA team using modern encryption standards, including secure file transfer protocol.

### 2.12 Review study datasets when received

The IPD-MA team should compare the data received to the study codebook, protocol, and publications as soon as possible after receiving the data. At a minimum, the study team should verify that the sample size, number of cases, and the inclusion of key exposure and outcome variables in the shared dataset match existing documentation or the study team’s understanding of their dataset if documentation of changes to the codebook or protocol was not shared. In the research response to an EID where studies may be ongoing, as sharing for harmonization begins, study numbers are in flux and cannot be directly matched to publications. That said, the IPD-MA team can flag these differences, especially in the number of infected and noninfected, for the team’s review to ensure the most recent data were received.

### 2.13 Clean study data

Research and data collection in the context of an outbreak are challenged by resource limitations when frontline staff manage high patient loads, infection control procedures, and staff illness. Data cleaning for an IPD-MA of an emerging pathogen will be more labor intensive than IPD-MAs for well-characterized exposures. To facilitate the data-cleaning process, the IPD-MA’s harmonization team should set up a series of calls to review the distribution of key variables with the primary study team. Having at least the study principal investigator (PI) and data manager on these calls helps identify and resolve data-cleaning issues efficiently. These calls represent a significant time investment for data-contributing groups. Still, they can also be seen as a type of benefit sharing where participation in the IPD-MA helps investigators identify and remedy data-cleaning issues in their datasets. Study datasets often include text fields in multiple languages, with abbreviations and shorthand references according to local standards. The IPD-MA team will need to include at least one and ideally several ID physicians who are fluent in the languages of the initial studies to appropriately interpret these data, either manually or through assisting the team in developing a natural-language-processing-based algorithm to characterize the textual data appropriately. The resources needed for this should be considered proactively as the heterogeneity in text representations of similar concepts is not insignificant, and both physician time and machine learning algorithm development require financial support.

### 2.14 Collect study-level and IPD-MA-level metadata

Study-level metadata describe the selection of the study population, within- and across-study differences in exposure and outcome ascertainment between study groups (e.g., symptomatic and asymptomatic) and over time, and the measurement of important confounders. Without well-characterized metadata, IPD are uninterpretable and cannot be harmonized or analyzed. In addition, study- and subject-level metadata are central to imputing study- and participant-level data.^62^ While not explicit in the dataset, infection or symptom status may be set at the study level through inclusion criteria when only infected or symptomatic participants are recruited. These study-level metadata must be documented to ensure appropriate harmonization and interpretation of the data. Discovery and descriptive metadata are integral to implementing the FAIR principles, and several tools exist for defining machine-actionable metadata.^63^ ^,^ ^64^

While metadata can be extracted from published protocols, in most cases, the protocols will not describe all necessary metadata. Protocols and their implementation will differ importantly over time and across sites as more accurate diagnostics are adopted or as the understanding of key confounders changes as the science related to the EID evolves. In the research response to an emerging pathogen, studies may change screening and diagnostic algorithms and laboratory and data collection protocols to incorporate the most recent diagnostics or information on transmission and the relation between symptoms and infection. To appropriately document these changes for inclusion in the outcome models, the IPD-MA team should work with data contributors to develop a metadata survey that captures within- and cross-study differences in study inclusion criteria, diagnostic tools, laboratory protocols, exposure, confounders, and outcome ascertainment.^19^ The metadata survey’s development, completion, and analysis represent a significant investment of time that should be included when discussing the time required for IPD-MA participation with study investigators. The desire for comprehensiveness must be balanced with the time investigators have available to complete the metadata survey and the number of metadata variables that can be included in desired analyses (e.g., the number of metadata variables would not exceed the number of participant-level variables in any model). We describe the different types of metadata and special metadata concerns in the research response to an EID in Table 1.

**Table 1:** Different types of metadata needed for an IPD-MA of an EID

Type	Definition	How to collect	How to disseminate
IPD-MA-related metadata	Harmonization rules and related code that describe how study-level definitions are transformed for harmonization to the master data dictionary, and other information that describes how the different datasets are combined into a pooled one. How measurements were defined at the IPD-MA level (e.g., does it represent a harmonized concept?).	Determined through interactions with working groups. Metadata survey provided to participating studies. Data extraction from publicly available documents (e.g., publications, protocols).	Publication in an open-access repository for text or code (e.g., Zenodo, GitHub).
Study-level metadata for each dataset	These include the study location, principal investigators and data management team, PICOT, inclusion and exclusion criteria, follow-up procedures, exposure, outcome, and covariate definitions and ascertainment procedures, testing algorithms and quality control procedures, study protocol, and codebook. While these are generally time fixed in IPD-MAs of NCDs or well-characterized IDs, they will be time varying in the research response to an EID, necessitating different collection procedures.	Metadata survey provided to participating studies that allows respondents to specify dates when study-level metadata changed during the epidemic (e.g., when a new diagnostic was introduced). Data extraction from publicly available documents (e.g., publications, protocols) can be used to supplement a survey but are unlikely to provide precise dates when key protocol changes were introduced.	Publication in an open-access metadata catalog (e.g., European Clinical Research Infrastructure Network (ECRIN) trials metadata repository) or open-access repository (e.g., Zenodo) if data contributors agree.
Participant-level metadata	Metadata associated with specific file formats (e.g., DICOM); participant-level metadata needed to characterize related data (e.g., phenopacket), study participants’ eligibility criteria (when different from study eligibility criteria).	Produced automatically as part of data capture or manually through standardization or creating linkages between data types.	Depends on the sensitivity of the data type.

*Abbreviations*: DICOM, Digital Imaging and Communications in Medicine; EID, emerging infectious disease; IPD-MA, individual participant data meta-analysis; PICOT, patient, intervention, comparison, outcome, and time.

## 3 Harmonize participant-level data

Harmonization represents the most resource-intensive part of any IPD-MA for both the core management team and data contributors. Harmonization is even more difficult for EIDs where data capture standards may not yet exist and are unlikely to be applied. Convening study leads early in the epidemic can facilitate the coordinated development of standard cohort protocols, eCRFs, and exposure and outcome definitions, as was the case with the WHO-ISARIC eCRF, which was made freely available early in the COVID-19 pandemic.^23^ While these efforts may help improve coordination, retrospective harmonization of disparate CRFs, variable names, data formats, and units across divergent enrollment and follow-up protocols is unavoidable. As with the emergence of ZIKV, where dengue-virus-related cohort studies were the first to identify and characterize the ZIKV outbreak,^43^ when available, ongoing cohorts that preexist the emerging pathogen are an essential resource for an IPD-MA of an emerging pathogen, even though the study protocols differ importantly from those of more recent studies. While retrospective harmonization of participant-level data from preexisting cohorts is labor intensive, these data provide the individual-level longitudinal data needed to understand heterogeneous transmission and progression.^65^

For an IPD-MA of an EID, harmonization can take between 3 months to one or more years,^66^ so the IPD-MA core team will need to negotiate early access to the data to begin harmonization. Contractual assurance that data will be kept confidential, that data will not be used for analyses other than those described in the protocol, and that publications will not be submitted outside of agreed-upon timelines can help build trust and alleviate concerns about sharing. The harmonization process is also an opportunity to identify studies that evaluated a particular aspect of the manifestations of the EID or that used more sophisticated diagnostic tools, which can lead to additional analyses that focus specifically on these areas.

### 3.1 Quantify the harmonization potential

Harmonization potential, defined as whether the study-specific variable definitions, levels, and ascertainment are similar enough to the master variable to be harmonized, can be determined through a combination of qualitative (expert consultation) and quantitative (review of distributions, cross-study correlation, related metrics) methods. In an IPD-MA conducted in response to an EID, the evaluation of harmonization potential is iterative as new data are produced and new findings emerge. Re-evaluation of harmonization potential will lead to adjustments in the types or ranges of proposed key variables. Determining what is acceptable or not for harmonization or heterogeneity-related quality assessments is essential to harmonization-related tasks during the research response to an emerging pathogen. Assessing the harmonization potential may also include Consortium-wide discussions about whether dataset limitations warrant the investment of time and effort needed to harmonize the data.

### 3.2 Decentralized versus centralized harmonization

Outside of the research response to emerging pathogens, consistency in exposure and outcome definitions, diagnostic criteria and assay sensitivity, use of standardized units, and general agreement on confounders that must be measured mean that studies often harmonize their variables for the IPD-MA. Given the high level of cross-study heterogeneity in variable definitions and measurement methods, when conducting an IPD-MA in response to an emerging pathogen, retrospective harmonization by individual teams should be considered as a last resort. Harmonization-related decisions must be made at the group level and clearly described in text and code with clear versioning, as they may change as the EID is better understood. Using systems that link documentation and code, such as markdown documents, usable in different software languages, or harmonization-related software (e.g., Opal),^67^ that connects the description of the harmonization decision to the code that implements those decisions, is recommended. This allows seamless updating of the harmonized datasets as the team changes their opinion or finds inconsistencies in how harmonization rules are applied. When investigators are unwilling or unable to share de-identified datasets for centralized harmonization, they can conduct the harmonization in-house using a harmonization SOP and code template. The IPD-MA harmonization group can then incorporate the documentation of harmonization-related decisions from that team’s application of the harmonization code.

### 3.3 Describe and confront variation in study quality

Restricting study inclusion by quality is a contentious topic. Some markers of study quality will not be well understood at the beginning of the research response to the EID. For example, selection bias, including relating enrollment or follow-up to infection and its sequelae, may be identified as the understanding of the pathogen evolves. In the research response to the 2015–2017 ZIKV pandemic, initial cohort enrollment was often limited to symptomatic pregnant women, so the probability of entering the study was related to the probability of experiencing the outcome, a form of selection bias.^68^ In some studies, the infants of women who were symptomatic for ZIKV were followed and assessed more closely than those of women who were not symptomatic, such that the probability of the exposure was related to the measurement error in the outcome.

RoB assessment, the assessment of studies’ internal validity, is an integral part of any meta-analysis and should be conducted before and following the receipt of IPD and assessed at the study and participant level.^14^ A recent systematic review of IPD-MA of test accuracy and clinical risk prediction models found that many IPD-MAs fail to conduct RoB assessments or to conduct and report on the assessments adequately.^14^ The IPD-MA presents an opportunity to apply RoB assessment consistently across studies and to assess how RoB affects results quantitatively.

Study quality may be impacted by resource availability, and the exclusion of lower-quality studies may result in the exclusion of researchers from less-resourced countries. For surveillance-based studies or studies limited to only cases or participants who have experienced an adverse event, the analyses must account for the lack of a control group. Determining study quality before receiving the data may not be possible. The newly created consortium for the EID may set specific criteria for inclusion, such as recruitment of a control group, use of virologic rather than serologic assays, or inclusion of key confounders before collecting and reviewing data. The importance of equitable inclusion of research from high and low-and-middle-income (LMIC) settings should be considered and can be addressed by flexibility in data quality requirements or providing support to improve data quality.

## 4 Statistical concerns

Novel statistical approaches are needed to address heterogeneity, complex correlated forms of measurement error, and different patterns of missingness in IPD-MAs conducted in the research response to emerging pathogens. Bayesian methods facilitate the inclusion of subject matter expertise to inform the estimation (e.g., through informative priors) of key parameters and better characterize spurious and informative sources of heterogeneity. Although harmonization may help address important challenges when pooling heterogeneous cohort data, additional steps are needed to facilitate their analysis. Key statistical challenges in IPD-MA of EID and recent advances, summarized in Table 2 and described briefly below, are selection bias, measurement error, and study- and subject-level missingness, as well as studies without a clear population at risk (denominator). While statistical methods exist to address these issues separately, in the context of an IPD-MA of an emerging pathogen, these challenges need to be addressed simultaneously. Several methodological advances to facilitate accurate cross-study inference in the research response to an emerging pathogen were developed during the research response to COVID-19.

**Table 2:** Key statistical issues to address in an IPD-MA of an EID

		Potential approaches and needed
Statistical challenge	Description	extensions to existing methods
Measurement error (spurious bias in exposure, outcome and covariate ascertainment, follow-up and comparators)
Combining IPD and AD	Not all studies will provide IPD or will provide IPD in the desired granularity or timeline. Studies from which only AD are available may not have the same point estimates, exposure, outcome, or covariate estimates, or ascertainment of those measures.	Implement two-stage meta-analysis that uses participant-level data where available and AD where not available. Implement iterative Bayesian updating as new data and knowledge emerge. The Bayesian framework offers flexible modeling structures, allowing the integration of information in the form of informative priors where only aggregated information is available, while addressing other statistical issues simultaneously.
Between- or within-study heterogeneity in diagnostic algorithms and variable definitions	Diagnostic tools (and their availability, use, and interpretation), diagnostic algorithms, and ascertainment and definitions of the exposure, outcome, and confounders evolve with the emergence of the new pathogen and/or the epidemic stage. Measurement error due to the use of new/non-validated diagnostic tools could cause misclassification of the outcome/exposure.	Create a panel to provide quantitative estimates of the differences in sensitivity and sensitivity of different diagnostic tools. Include these correction values as regression parameters. Identify sources and timeline for the changes in ascertainment to account for this heterogeneity in the imputation and outcome models. Conduct sensitivity analyses to assess the impact of variation in diagnostic tool accuracy on model estimates.
Missing data		
Study-level missingness	Important confounders not measured by the study or not measured at the beginning of the study.	Two-stage meta-analysis that uses IPD from fully adjusted models where available and point estimates and their uncertainty where not available. Implement Bayesian model updating.
Participant-level missingness	Loss to follow-up and absence of data (can be at random or not at random).	Participant-level data can be imputed within studies using data from those studies and known associations from related studies.
Lack of a control group (case-only data) or non-representative controls	Studies that use surveillance data and cohort studies with limited funding may restricted to participants with certain criteria.	Use the inverse probability of selection weights to compare to external control groups.
Differences in timing and spacing of visits and duration of follow-up	Funding delays, insufficient funding, and feasibility concerns may influence the time of recruitment and follow-up. Also, characteristics of the pathogen (e.g., seasonality) and the evolving understanding of the pathogen (e.g., incubation period, symptoms, body systems affected, and sequelae) will lead to variations in the timing, spacing, and duration of follow-up within and across studies.	Both selection into the study or ascertainment of the outcome that are related to the probability of outcome (selection bias) and measurement error related to differences in covariate ascertainment can be considered missing data problems and addressed through imputation. One-stage participant-level imputation with random effects at the participant and study-level to account for clustering within individuals and studies while leveraging information across individuals within studies and across studies.

*Abbreviations*: AD, aggregate data; EID, emerging infectious disease; IPD, individual participant data.

### 4.1 Selection bias, measurement error, and missingness

Heterogeneity in exposure, outcome, and covariate ascertainment, follow-up, and comparators is a major concern in IPD-MAs of emerging pathogens.^19^ The sources of heterogeneity, degree of the variability in effect estimates due to heterogeneity, and implications for the interpretation of results need to be addressed. In the research response to an EID, the lack of available or accurate diagnostic tools or lack of specialists at some sites may lead to higher levels of measurement error in the exposure or outcome than expected in IPD-MAs of well-described exposures and outcomes. Given the lack of clarity on infection and its sequelae, selection into the study of the EID is likely related to the probability of infection and its detection, especially in the early stage of an outbreak. Hence, the widely used but difficult-to-defend assumption that measurement error is often non-differential and that ascertainment of the exposure or outcome is unrelated to the probability of exposure or disease, used by researchers to ignore measurement error, becomes even more untenable than usual. Both selection bias and measurement error at the design stage are related to the lack of understanding of the EID and can be conceptualized as a missing data problem that can be addressed through multiple imputation, if sufficient study- and participant-level data are available to develop the imputation models.

Addressing study-level missingness and the complex forms of measurement error in IPD-MAs of EIDs, where the probability of the exposure is related to the outcome, are outstanding challenges. Although many statistical methods are available to address missing data, measurement error, and between-study heterogeneity separately, identifying and jointly resolving these spurious sources of variability is much more difficult. Additional challenges arise when the research question aims to identify participant-level effects of disease infection, treatment strategies, or policy decisions. Recent advances include novel Bayesian methods for addressing informative missingness^62^ and measurement error using informative priors from public studies or expert opinion in the context of IPD-MAs.^69^ ^,^ ^70^ The correlated nature of the missingness (i.e., patterned missingness due to lack of information about the pathogen) in IPD-MAs of EID limits the use of existing imputation approaches based on the “missing at random” or missing “not completely at random” assumptions. Hence, imputation should be undertaken and interpreted carefully.

### 4.2 Studies with no or no clear denominator

Studies conducted in the research response to an emerging pathogen may be limited to individuals infected with the disease. Similarly, for surveillance-system-derived datasets, the population at risk, the source population for cases, may be difficult or impossible to define. The absence of any or a well-specified denominator limits or precludes the ability to use these studies to estimate risk or change in risk across populations or time and limits the external validity of study findings.^71^ Surveillance-derived datasets may collect detailed information on the infected population and little-to-no information on the population at risk. Last, inclusion into the surveillance dataset (i.e., routine reporting) is correlated with health-seeking behavior, age, access to healthcare, socioeconomic and minority status, among other factors, which can create selection bias.^72^ When pooling case-only data with studies that include both an exposed and an unexposed group in an analysis that estimates the risk of the outcome, researchers need to estimate the population at risk for these studies and design imputation models for subject-level variables that are not recorded for these populations.

### 4.3 Methods used to address heterogeneity by COVID-19 IPD-MAs

In Table 3, we summarize how longitudinal COVID-19 IPD-MAs that obtained IPD by contacting authors (i.e., did not only utilize published data), identified through a rapid systematic review,^33^ addressed the statistical issues detailed above, including study- and participant-level missingness, selection bias, loss to follow-up, and measurement error. Out of the six published longitudinal COVID-19 IPD-MAs identified at the time of submission, three IPD-MAs mentioned approaches to address missing data.^73^ ^–^ ^75^ In one IPD-MA, the authors imputed missing outcome data and conducted case-wise deletion for missing covariate data.^75^ Another IPD-MA conducted “best-worst” and “worst-best” sensitivity analyses to assess the impact of missing participant-level data.^73^ In the last IPD-MA, the authors deemed that imputation of missing data was not required for the main predictors.^74^ Three IPD-MAs detailed how they addressed loss to follow-up,^73^ ^,^ ^75^ ^,^ ^76^ with one imputing missing outcome data,^75^ another using trial results reported at maximum follow-up for all outcomes,^73^ and the third conducting intention-to-treat analysis of the RCTs and non-randomized trials.^76^ One IPD-MA that included two RCTs and one non-randomized trial assessed the risk of selection bias by conducting a sensitivity analysis that excluded the non-randomized trial.^76^ Another IPD-MA also addressed selection bias by conducting a sensitivity analysis excluding the largest study in the IPD-MA.^77^ A third IPD-MA performed subgroup analysis assessing trials at low compared with at high RoB and trials without and with potential conflicts of interest to assess selection bias.^73^ In terms of confronting measurement error, one IPD-MA repeated the primary analysis on subsets of the data based on sampling site, data quality, and sampling frequency, and accounted for residual and random errors.^73^ Another IPD-MA assessed the risks of systematic bias using the Cochrane RoB tool version 2, conducted trial sequential analyses to control for false -positive results (i.e., type I error), and adjusted the thresholds for statistical significance to account for the risk of unmeasured confounding.^78^ Two IPD-MAs restricted included studies by study quality,^75^ ^,^ ^78^ one of which only included “high-quality” RCTs but did not specify how quality was assessed.^75^ The other IPD-MA conducted two separate quality assessments and only included studies with a quality score “1” or “2” from either quality assessment.^78^

**Table 3:** Longitudinal COVID-19-related IPD-MAs approach to addressing study quality and missingness

					Study-level			
				Exclusion	and			
				related	participant-			
Author,			Study	to study	level	Selection	Loss to	Measurement
year	Title	Objective	types	quality	missingness	bias	follow-up	error
Gastine, 2021^78^	Systematic review and patient-level meta-analysis of SARS-CoV-2 viral dynamics to model response to antiviral therapies	To identify serial viral loads with time in people with COVID-19 to describe and model the viral load trajectory	Case reports, case series, and clinical trials	Yes—two separate quality assessments were applied to each dataset and rated on a three-point scale, with 1 being highest quality. Only those with quality rating “1” or “2” from either quality assessment were included in different analyses.	NR	NR	NR	− Repeating the primary analysis on subsets of the data based on sampling site, data quality, and sampling frequency− Residual and random errors considered in statistical models
Harwood, 2022^77^	Which children and young people are at higher risk of severe disease and death after hospitalization with SARS-CoV-2 infection in children and young people: A systematic review and individual patient meta-analysis	To identify which children and young people were at increased risk of severe disease or death in those admitted to hospital with SARS-CoV-2 infection	Cohorts or other longitudinal observational studies or case series	No—authors acknowledge that the risk of bias assessment shows that the included studies are of low quality	NR	Sensitivity analysis excluding the largest study	NR	Not reported
Korang, 2022^73^	Vaccines to prevent COVID-19: A living systematic review with Trial Sequential Analysis and network meta-analysis of randomized clinical trials	To assess the effectiveness and safety of COVID-19 vaccines through analyses of RCTs	RCTs	No	Sensitivity analyses using “best-worst” and “worst-best” analysis to assess the impact of missing data	Performed subgroup analysis assessing trials at low compared with high risk of bias and trials without and with conflicts of interest	Used trial results reported at maximum follow-up for all outcomes	Assessed the risks of systematic errors through bias risk assessment, conducted trial sequential analyses to control random errors, and adjusted the thresholds for statistical significance to control the risks of random errors
Simmons, 2020^76^	Sofosbuvir/daclatasvir regimens for the treatment of COVID-19: an individual patient data meta-analysis	To determine whether sofosbuvir/daclatasvir-based regimens improve clinical outcomes of patients hospitalized with moderate or severe COVID-19	RCTs and non-randomized intervention studies	No	NR	Sensitivity analysis excluding non-randomized trials	ITT analysis	Not reported
Subramaniam, 2022^74^	Characteristics and outcomes of patients with frailty admitted to ICU with coronavirus disease 2019: An individual patient data meta-analysis	To evaluate the characteristics and outcomes across the range of frailty in patients admitted to the ICU with COVID-19	Prospective and retrospective cohort studies	No	The authors deemed imputation not necessary for the main predictors	NR	NR	NR
Troxel, 2022^75^	Association of Convalescent Plasma Treatment With Clinical Status in Patients Hospitalized With COVID-19: A meta-analysis	To understand the safety and potential benefit of convalescent plasma to treat hospitalized patients with COVID-19	RCTs	Yes—mentions that only “high-quality” RCTs are included	−Imputation for missing outcome data − Case-wise deletion for missing covariate data	NR	Imputation for missing outcome data	NR

*Abbreviations*: ITT, intention to treat; NR, not reported.; RCTs, randomized controlled trials.

## 5 Ethical, legal, and technical considerations

The transfer, management, and use of data for IPD-MAs should follow the applicable standards and approvals of each country/region of data origin. These may include rules and regulations governing personal data protection, clinical trials, research, and human rights. Investigators should be aware of their local requirements and ensure they are met.

### 5.1 Broad consent for future use

Obtaining informed consent from research participants to reuse data for subsequent analyses is a robust approach to protecting the interests of participants in data reuse initiatives, including IPD-MAs. That said, most ethics review committees (ERCs) do not require primary studies to seek broad consent for future use when sharing participant-level data for an IPD-MA with the same research questions as the primary study that did seek informed consent. The Cochrane Collaboration Guidance states that when the IPD-MA uses de-identified data for the same purposes for which the data were originally collected, even when future use was not included in the study’s ICFs, re-consent of research participants is not required.^79^ Where studies did not include a provision for future use and the study’s ERC does not recognize the practice of using de-identified data from the study for an IPD-MA with the same objectives as the original study, investigators can apply for a waiver of consent. A waiver of consent may be supplied by the study’s ERC or the regional or national ERC if there is a clear public health rationale for the IPD-MA, as would be the case in the research response to an EID, or the risks related to recontacting study participants are greater than the risks related to sharing their data for the IPD-MA.

### 5.2 Surveillance data

Surveillance data are collected as part of the public health response to an emerging pathogen and generally include data from individuals who did not provide their informed consent for participation in a research study. EU privacy and data protection laws make provision for data processing for public health purposes provided certain conditions are satisfied (35 General Data Protection Regulation 2016/679), and most countries have similar laws in place to facilitate data reuse for the public health response to emerging pathogens.

### 5.3 Inclusion of vulnerable populations

The transmission and progression of an emerging pathogen may differ significantly across populations because of social factors, differences in immune response, or the distribution of other risk factors. Vulnerable populations are often excluded from research (e.g., emergency room patients, pregnant individuals, children, indigenous and other racialized groups, incarcerated populations, and documented and undocumented immigrants, migrants, and refugees). These populations are especially important to include in an IPD-MA of an emerging pathogen because differences in immunity, disease spread, and access to preventative measures, testing, and care mean that interventions developed for the general population may not be appropriate or sufficient.^80^ As these populations may be more susceptible to coercion, their inclusion in the IPD-MA warrants additional oversight from representatives of the population and through an international, national, and or local ERC review, wherever possible, that adequately represents the vulnerable populations.^81^ Research found that COVID-19 studies often excluded measuring or reporting on social factors associated with infection and disease.^80^ ^,^ ^82^ The IPD-MA team can consider using the PROGRESS (place of residence, race/ethnicity/culture/language, occupation, gender/sex, religion, education, socioeconomic status, and social capital) to ensure consideration of social determinants of vaccination, infection, and disease in imputation and outcome models.^83^ While studies included in the IPD-MA may focus on the risk of including vulnerable populations (e.g., lack of adequate time to engage the population), the risks related to excluding vulnerable populations in primary studies and subsequently in evidence synthesis in the research response to an EID should be considered along with the risks related to their inclusion.

### 5.4 Benefit sharing

In addition to joint-authorship and citation of primary studies’ datasets, the IPD-MA team should ask partner studies to identify their priorities for benefit sharing and work with partner studies to seek funding to support benefit sharing. Types of benefit sharing might include access to skill building in data stewardship or complex analyses, including IPD-MA, well-structured mentorship programs, or support for masters’ and doctoral students. Depending on the geographic area where the pathogen has emerged, the IPD-MA can create power imbalances in the research team when data are collected in under-resourced settings and managed and analyzed by researchers in high-resource settings. Investing in capacity building throughout the IPD-MA process and working with funders to establish centers for excellence for data synthesis and IPD-MA in settings that are traditionally underrepresented in the field of IPD-MA can help address these long-term imbalances. Study authors should be meaningfully engaged in research priority setting during and after the epidemic to ensure that research and development investments benefit the communities that participated in the research.^84^

### 5.5 Community engagement

To support the use of IPD-MA findings and the sharing of potential health benefits with the communities where data originate, IPD-MA teams should consider efforts to engage source communities. Community engagement, while considered best practice for IPD-MAs,^85^ is often an afterthought.^86^ The form that community engagement takes depends on the specific objectives of the IPD-MA. Community engagement should relate to the objectives of the IPD-MA and facilitate the dissemination and update of findings. For example, given the high levels of uncertainty in both the exposure and outcome assessment, and the sensitivities related to delivering uncertain information on adverse birth or developmental outcomes to pregnant women, the ZIKV IPD Consortium IPD-MA developed a qualitative study in three of the countries most affected by ZIKV to ascertain pregnant women’s preferences around risk communication in the presence of uncertainty.^87^

### 5.6 Identifying and applying community-developed data capture or exchange standards

While not essential for data analysis, harmonization to an internationally recognized standard for data and metadata facilitates the reuse of the IPD-MA dataset beyond the IPD-MA itself, pending data contributor agreement, and an ethical review of provisions for future use. Capturing FAIR-by-design data can promote machine readability, enable automated metadata extraction, and support long-term discovery and integration into knowledge graphs, dashboards, and cross-study analyses, including IPD-MAs. The training needed to apply these standards presents a particular challenge for clinical epidemiological data from observational studies where such standard ontologies (e.g., SNOMED-CT, Logical Observation Identifiers Names and Codes [LOINC], CDASH) are infrequently implemented.^47^ ^,^ ^51^ ^,^ ^88^ Standards may not be readily available at the start of the epidemic or outbreak, which further complicates their uptake. In COVID-19 response, ISARIC’s work to rapidly publish an open-source eCRF for COVID-19 data collection, based on CDISC standards, greatly facilitated the standardization of data collection.^23^

## 6 Discussion

This review provides an overview of the specific project management, ethical, data sharing, cleaning, harmonization, and analysis concerns and related recommendations when conducting an IPD-MA of an EID. Challenges specific to an IPD-MA of an emerging pathogen and a way forward are summarized in Table 4. In the research response to ZIKV, ongoing or published IPD-MAs are maximizing the utility of data on the characteristics of affected children and the spectrum of manifestations.^1^ ^,^ ^20^ ^,^ ^53^ In the global response to COVID-19, leveraging existing participant-level data and connecting different data types at the participant level for individualized diagnosis and prognosis made significant contributions to the evidence base.^36^ ^,^ ^89^ ^,^ ^90^

**Table 4:** Additional challenges faced in the conduct of an IPD-MA of an emerging pathogen and a way forward

	Challenge	Suggested approach
Barriers to data sharing	Investigators are clinicians on the front lines of the pandemic.	Prioritize the health of investigators and the populations they serve. Set realistic expectations.
	Limited time for relationship building to foster trust needed for data sharing.	Lead with transparency and inclusion. If possible, host an in-person meeting to facilitate future communication.
	Competing priorities and lack of time to clean data, complete their own publications, or prepare data for IPD-MA.	Consider two-stage IPD-MA to allow for heterogeneous timelines.
	Need to publish their own findings.	Establish clear agreements about the publication of IPD-MA findings.
	Governments may try to suppress data collection or sharing for fear their country will be targeted with control measures.	Support investigators in challenging situations.
	Most pathogens emerge in LMIC, teams that conduct IPD-MAs often based in high-income countries, which may lead to an imbalance in power.	Prioritize capacity building in the conduct of IPD-MAs if of interest to investigators.
Barriers to harmonization	High level of heterogeneity in how and what variables collected across studies and across time within studies because studies evolve with the understanding of the pathogen.	Establish an expert’s committee to oversee harmonization decisions. Update harmonization decisions as needed.
	Disagreements on which variables to include in the analysis can lead to too many variables included in harmonization.	Set realistic expectations to ensure a harmonized dataset can be delivered on time.
	Codebook and protocol may have changed over time and no longer correspond to the data.	Ensure proper versions of codebook, protocol, and dataset received. Review questions with the team that collected data.
Barriers to analysis	Difficult to avoid selection bias since symptoms are unknown or partially known.	Account for selection bias and measurement error.
	High levels of measurement error in the exposure in the absence of a gold standard diagnostic.	Conduct participant- and study-level imputation and address high levels of measurement error.
	Not clear what outcomes are or how to measure them	Update the model as new findings emerge.
	Lack of consensus on a minimal set of confounders.	Conduct extensive sensitivity analyses.
	Lack of metadata.	Conduct a metadata survey.
Barriers to risk communication	A high level of variability should lead to caution in interpreting findings.	Engage the community to understand preferences around risk communication.

*Abbreviations*: IPD-MAs, individual participant data meta-analyses; LMICs, low- and middle-income countries.

Despite guidance and mandates from funding organizations, significant barriers to sharing and analyzing participant-level population and clinical-epidemiological data have limited the types of data shared and delayed critical analyses that depended on data sharing.^30^ ^,^ ^31^ ^,^ ^58^ In the context of a novel emerging pathogen, there is no gold standard diagnostic test nor a clearly defined minimal set of confounders for adjustment. Study quality can be expected to improve as the epidemic progresses, but imposing thresholds for study quality as a criterion for inclusion in the IPD-MA may have the unintended effect of excluding datasets from researchers in less-resourced settings or reducing the number of cases in the dataset for fast-moving epidemics.

Considering the six COVID-19 IPD-MAs of longitudinal studies that we identified in a rapid systematic review, neither of the two COVID-19 IPD-MAs that addressed missing data used multiple imputation although one IPD-MA of RCTs used imputation to account for loss-to-follow-up. Several studies accounted for differences in study quality through sensitivity analyses, including restricting the analyses based on study quality. Evaluating the impact of advanced methods for addressing measurement error and missingness on IPD-MA findings may support the refinement and uptake of these methods.

**Figure 2:**
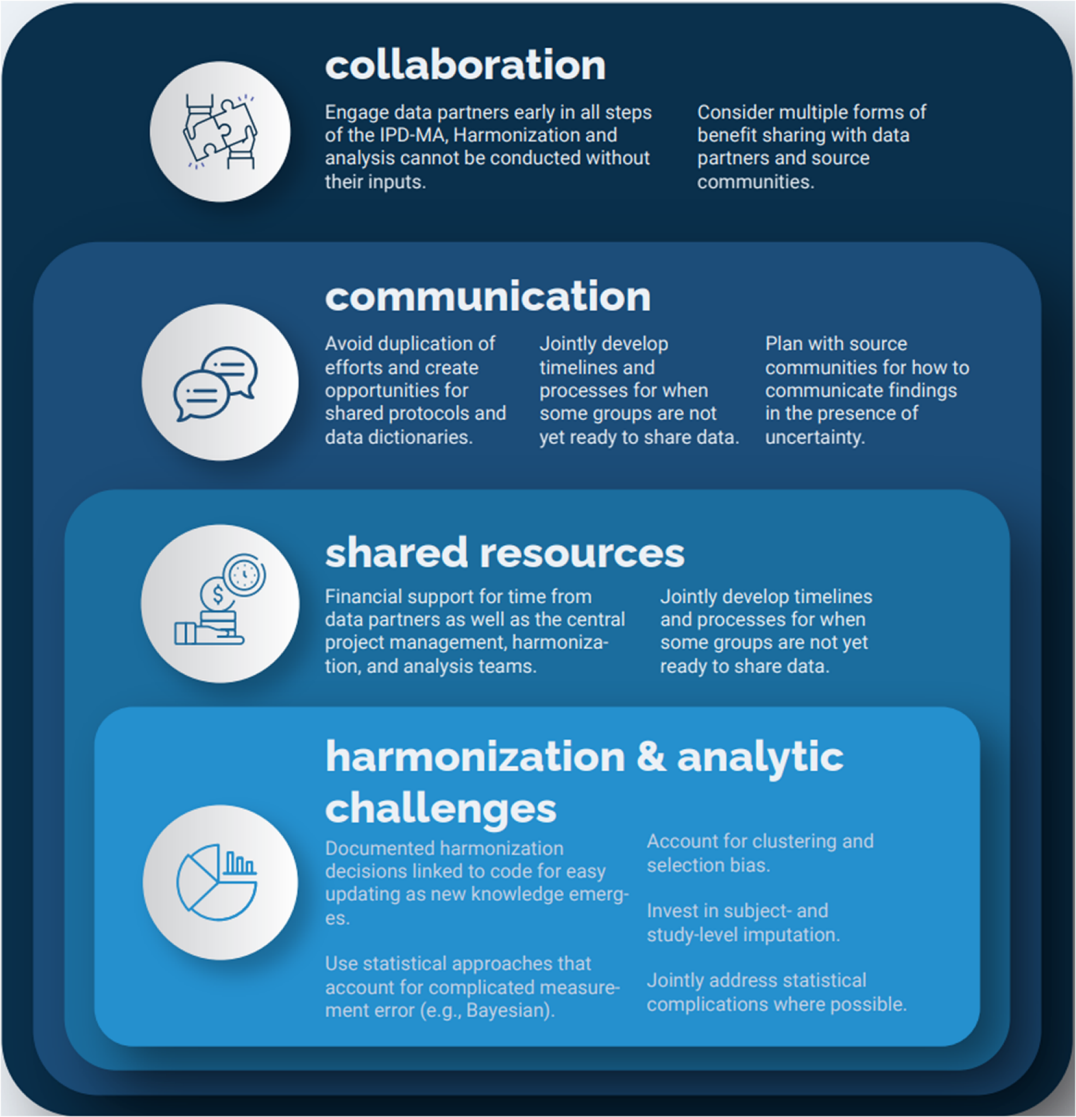
Core principles for the conduct of an IPD-MA of an emerging pathogen.

IPD-MAs require open communication between a diversity of experts built on trust and a clearly defined, shared goal. In the research response to an emerging pathogen, the rationale for such collaborations is well established. Still, there are several challenges that should be confronted early on to ensure the project’s timely execution. New strategies are needed to train investigators in data stewardship, including implementing the FAIR principles,^29^ and in conducting IPD-MAs to facilitate the rapid reuse of existing data. As shown in Figure 2, IPD-MAs conducted in the research response to an emerging pathogen must be rooted in collaboration. Communication follows collaboration and will inform both the IPD-MA and future IPD-MAs given that the same groups of researchers are likely to be on the front lines of the next pandemic. Shared resources help to build equity in the research response to EIDs and support communication and collaboration. Last, harmonization and analysis must be rooted in shared resources, communication, and collaboration to ensure that the teams that led the primary studies colead the harmonization and analysis process.

## 7 Conclusion

IPD-MAs facilitate the research response to emerging pathogens by leveraging limited resources (investigator time, expertise, funding, test kits, case data) to make the most of existing data in a timely fashion. While AD-MAs provide more rapid evidence for policy during outbreak response, IPD-MAs may prospectively address research and data integrity issues that can lead to biased inference when data are not made available and evidence is scarce early in the outbreak or epidemic. The heterogeneity in study protocols, measurement, and confounder ascertainment complicates the harmonization and analysis of IPD in the research response to an emerging pathogen. Data sharing has evolved significantly since the Ebola^24^ and ZIKV epidemics, and the rapid establishment of consortia and platforms for data reuse in COVID response (e.g., ISARIC,^23^ CAPACITY-COVID^91^) has paved the way for PMAs in response to future EIDs. That said, the global response to COVID-19 is not immune to longstanding concerns related to PEARL data sharing barriers and the statistical challenges inherent in IPD-MAs of emerging pathogens. We have come a long way and have a long way to go.

## Data Availability

All relevant data are included in the article.
